# 4-Amino-3-(4-hydroxy­phen­yl)-1*H*-1,2,4-triazol-5(4*H*)-one

**DOI:** 10.1107/S1600536808039688

**Published:** 2008-11-29

**Authors:** Kai-Ge Shi, Guang Yang, Seik Weng Ng

**Affiliations:** a Department of Chemistry, Zhengzhou University, Zhengzhou 450052, People’s Republic of China; b Department of Chemistry, University of Malaya, 50603 Kuala Lumpur, Malaysia

## Abstract

The mol­ecule of the title compound, C_8_H_8_N_4_O_2_, is nearly planar, with a dihedral angle between the rings of 1.1 (1)°. Adjacent mol­ecules are linked into a layered structure by hydr­oxy–oxo O—H⋯O and triazol­yl–hydr­oxy N—H⋯O hydrogen bonds. Only one of the H atoms of the pyramidal amino group is engaged in building up the infinite layer. The second H atom of the amino group also shows hydrogen-bonding inter­actions, linking adjacent layers into a three-dimensional network.

## Related literature

For a synthesis of the title compound using CS_2_ as a reactant, see: Chande & Singh-Jathar (1998 ▶). This product was obtained unexpectedly in the present study.
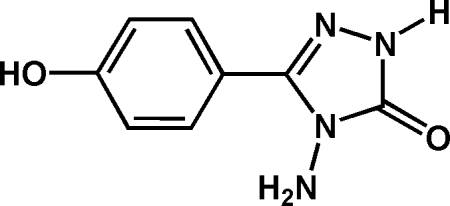


## Experimental

### 

#### Crystal data

C_8_H_8_N_4_O_2_
*M* *_r_* = 192.18
Triclinic, 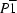

*a* = 6.534 (1) Å
*b* = 7.330 (1) Å
*c* = 9.804 (1) Å
α = 106.69 (1)°
β = 102.328 (9)°
γ = 106.712 (2)°
*V* = 407.7 (1) Å^3^
*Z* = 2
Mo *K*α radiation
μ = 0.12 mm^−1^
*T* = 295 (2) K
0.25 × 0.16 × 0.04 mm

#### Data collection

Bruker APEXII area-detector diffractometer
Absorption correction: none
3032 measured reflections
1434 independent reflections
1115 reflections with *I* > 2σ(*I*)
*R*_int_ = 0.017

#### Refinement

*R*[*F* ^2^ > 2σ(*F* ^2^)] = 0.039
*wR*(*F* ^2^) = 0.109
*S* = 1.03
1434 reflections
143 parameters
4 restraints
H atoms treated by a mixture of independent and constrained refinement
Δρ_max_ = 0.14 e Å^−3^
Δρ_min_ = −0.19 e Å^−3^

### 

Data collection: *APEX2* (Bruker, 2007 ▶); cell refinement: *SAINT* (Bruker, 2007 ▶); data reduction: *SAINT*; program(s) used to solve structure: *SHELXS97* (Sheldrick, 2008 ▶); program(s) used to refine structure: *SHELXL97* (Sheldrick, 2008 ▶); molecular graphics: *X-SEED* (Barbour, 2001 ▶); software used to prepare material for publication: *publCIF* (Westrip, 2008 ▶).

## Supplementary Material

Crystal structure: contains datablocks global, I. DOI: 10.1107/S1600536808039688/im2089sup1.cif

Structure factors: contains datablocks I. DOI: 10.1107/S1600536808039688/im2089Isup2.hkl

Additional supplementary materials:  crystallographic information; 3D view; checkCIF report

## Figures and Tables

**Table 1:** Hydrogen-bond geometry (Å, °)

*D*—H⋯*A*	*D*—H	H⋯*A*	*D*⋯*A*	*D*—H⋯*A*
O1—H1⋯O2^i^	0.86 (1)	1.78 (1)	2.633 (2)	175 (3)
N2—H2⋯O1^ii^	0.86 (1)	1.93 (1)	2.789 (2)	173 (2)
N4—H4⋯O2^iii^	0.87 (1)	2.24 (1)	3.077 (3)	163 (2)

Symmetry codes: (i) 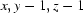; (ii) 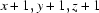; (iii) 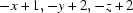.

**Fig. 1.**
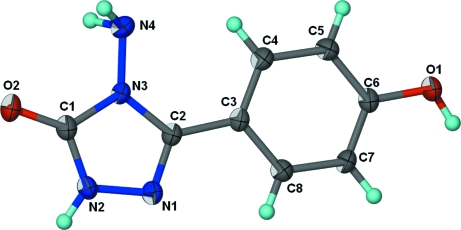
Thermal ellipsoid (Barbour, 2001) plot of C18H8N4O2 at the 70% probability level.

**Fig. 2.**
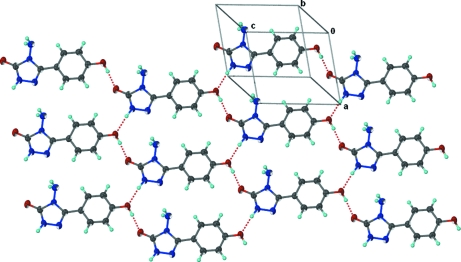
Thermal ellipsoid (Barbour, 2001) plot of the layered structure arising from O–Hhydroxy and N–Htriazolyl hydrogen bonds.

## supplementary crystallographic information

### Comment

In connection with our work on metal triazolates, we are interested in synthesizing 4-amino-bis(4-hydroxyphenyl)-1,2,4-triazole. The synthesis of this triazole yielded the title compound as an unexpected product. A specific procedure for the synthesis of the title compound is reported in the literature to start from carbonyl sulfide and 4-hydroxybenzohydrazide in potassium hydroxide to give a precursor that was subsequently reacted with hydrazine (Chande & Singh-Jathar, 1998).

### Experimental

4-Hydroxybenzoic acid (2.76 g, 0.02 mol) and 80% hydrazine hydrate (1.55 g, 0.02 mol) were heated in a sealed tube at 439 K for three days. After cooling to room temperature, the mixture was centrifuged. The resulting white solid was suspended in water, and 6*M* hydrochloric acid was added until the pH was 3. The white product was collected and recrystallized from a DMSO–water mixture(10:1) to afford colorless crystals in 3% yield. CH&N elemental analysis. C 49.58 (calc. 49.99), H 4.19 (found 4.20), N 29.25% (29.15%).

### Refinement

Carbon-bound H atoms were generated geometrically (C–H 0.93 Å), and were included in the refinement in the riding model approximation, with *U*(H) set to 1.2*U*_eq_(C). The amino and hydroxy H atoms were located in a difference Fourier map, and were refined with distance restraints of N–H = O–H = 0.85±0.01 Å; their temperature factors were freely refined.

### Crystal data

**Table:**

C_8_H_8_N_4_O_2_	*Z* = 2
*M**_r_* = 192.18	*F*_000_ = 200
Triclinic, *P*1	*D*_x_ = 1.565 Mg m^−^^3^
Hall symbol: -P 1	Mo *K*α radiation λ = 0.71073 Å
*a* = 6.534 (1) Å	Cell parameters from 986 reflections
*b* = 7.330 (1) Å	θ = 2.3–26.7º
*c* = 9.804 (1) Å	µ = 0.12 mm^−^^1^
α = 106.69 (1)º	*T* = 295 (2) K
β = 102.328 (9)º	Block, colorless
γ = 106.712 (2)º	0.25 × 0.16 × 0.04 mm
*V* = 407.7 (1) Å^3^	

### Data collection

**Table:**

Bruker APEXII area-detector diffractometer	1115 reflections with *I* > 2σ(*I*)
Radiation source: fine-focus sealed tube	*R*_int_ = 0.017
Monochromator: graphite	θ_max_ = 25.0º
*T* = 295(2) K	θ_min_ = 2.3º
φ and ω scans	*h* = −7→7
Absorption correction: None	*k* = −8→8
3032 measured reflections	*l* = −11→11
1434 independent reflections	

### Refinement

**Table:**

Refinement on *F*^2^	Secondary atom site location: difference Fourier map
Least-squares matrix: full	Hydrogen site location: inferred from neighbouring sites
*R*[*F*^2^ > 2σ(*F*^2^)] = 0.039	H atoms treated by a mixture of independent and constrained refinement
*wR*(*F*^2^) = 0.109	*w* = 1/[σ^2^(*F*_o_^2^) + (0.0565*P*)^2^ + 0.1018*P*] where *P* = (*F*_o_^2^ + 2*F*_c_^2^)/3
*S* = 1.03	(Δ/σ)_max_ = 0.001
1434 reflections	Δρ_max_ = 0.14 e Å^−^^3^
143 parameters	Δρ_min_ = −0.19 e Å^−^^3^
4 restraints	Extinction correction: none
Primary atom site location: structure-invariant direct methods	

### Fractional atomic coordinates and isotropic or equivalent isotropic displacement parameters (Å^2^)

**Table:**

	*x*	*y*	*z*	*U*_iso_*/*U*_eq_	
O1	0.3462 (2)	0.3381 (2)	0.06978 (15)	0.0481 (4)	
O2	0.6706 (2)	1.2219 (2)	1.00449 (15)	0.0456 (4)	
N1	0.8848 (3)	1.0137 (3)	0.72942 (18)	0.0423 (5)	
N2	0.9057 (3)	1.1419 (3)	0.87015 (19)	0.0444 (5)	
N3	0.5523 (2)	0.9858 (2)	0.75552 (16)	0.0319 (4)	
N4	0.3171 (3)	0.9141 (3)	0.7256 (2)	0.0420 (5)	
C1	0.7075 (3)	1.1281 (3)	0.8902 (2)	0.0361 (5)	
C2	0.6672 (3)	0.9197 (3)	0.6610 (2)	0.0314 (4)	
C3	0.5739 (3)	0.7693 (3)	0.5056 (2)	0.0302 (4)	
C4	0.3450 (3)	0.6652 (3)	0.4264 (2)	0.0373 (5)	
H4A	0.2398	0.6910	0.4719	0.045*	
C5	0.2715 (3)	0.5241 (3)	0.2810 (2)	0.0402 (5)	
H5	0.1177	0.4571	0.2289	0.048*	
C6	0.4261 (3)	0.4820 (3)	0.2127 (2)	0.0342 (5)	
C7	0.6551 (3)	0.5851 (3)	0.2893 (2)	0.0387 (5)	
H7	0.7598	0.5593	0.2432	0.046*	
C8	0.7269 (3)	0.7262 (3)	0.4342 (2)	0.0386 (5)	
H8	0.8808	0.7942	0.4854	0.046*	
H1	0.450 (3)	0.301 (4)	0.043 (3)	0.067 (8)*	
H2	1.037 (2)	1.204 (3)	0.937 (2)	0.054 (7)*	
H4	0.294 (5)	0.881 (4)	0.801 (2)	0.076 (9)*	
H40	0.273 (4)	1.013 (3)	0.732 (3)	0.078 (10)*	

### Atomic displacement parameters (Å^2^)

**Table:**

	*U*^11^	*U*^22^	*U*^33^	*U*^12^	*U*^13^	*U*^23^
O1	0.0338 (8)	0.0580 (10)	0.0293 (8)	0.0155 (7)	0.0038 (6)	−0.0101 (7)
O2	0.0431 (9)	0.0556 (9)	0.0288 (8)	0.0213 (7)	0.0126 (6)	−0.0008 (7)
N1	0.0305 (9)	0.0498 (10)	0.0295 (9)	0.0122 (8)	0.0071 (7)	−0.0045 (8)
N2	0.0288 (9)	0.0540 (11)	0.0268 (9)	0.0114 (8)	0.0029 (7)	−0.0095 (8)
N3	0.0271 (8)	0.0396 (9)	0.0245 (8)	0.0135 (7)	0.0099 (6)	0.0032 (7)
N4	0.0295 (9)	0.0559 (12)	0.0343 (10)	0.0172 (9)	0.0124 (8)	0.0053 (9)
C1	0.0347 (11)	0.0406 (11)	0.0262 (10)	0.0156 (9)	0.0081 (8)	0.0025 (9)
C2	0.0301 (10)	0.0349 (10)	0.0259 (10)	0.0132 (8)	0.0097 (8)	0.0051 (8)
C3	0.0312 (10)	0.0325 (10)	0.0255 (10)	0.0135 (8)	0.0103 (8)	0.0061 (8)
C4	0.0309 (10)	0.0442 (12)	0.0320 (11)	0.0165 (9)	0.0111 (8)	0.0042 (9)
C5	0.0264 (10)	0.0472 (12)	0.0343 (11)	0.0120 (9)	0.0056 (8)	0.0023 (9)
C6	0.0339 (11)	0.0380 (11)	0.0246 (10)	0.0136 (9)	0.0075 (8)	0.0043 (8)
C7	0.0316 (11)	0.0476 (12)	0.0297 (11)	0.0153 (9)	0.0124 (8)	0.0019 (9)
C8	0.0276 (10)	0.0450 (12)	0.0310 (11)	0.0110 (9)	0.0067 (8)	0.0017 (9)

### Geometric parameters (Å, °)

**Table:**

O1—C6	1.368 (2)	C2—C3	1.470 (2)
O1—H1	0.861 (10)	C3—C4	1.389 (3)
O2—C1	1.247 (2)	C3—C8	1.394 (3)
N1—C2	1.308 (2)	C4—C5	1.381 (3)
N1—N2	1.381 (2)	C4—H4A	0.9300
N2—C1	1.331 (3)	C5—C6	1.383 (3)
N2—H2	0.860 (10)	C5—H5	0.9300
N3—C1	1.374 (2)	C6—C7	1.385 (3)
N3—C2	1.380 (2)	C7—C8	1.379 (3)
N3—N4	1.407 (2)	C7—H7	0.9300
N4—H4	0.868 (10)	C8—H8	0.9300
N4—H40	0.846 (10)		
			
C6—O1—H1	112.3 (18)	C4—C3—C2	124.64 (17)
C2—N1—N2	104.87 (15)	C8—C3—C2	117.37 (17)
C1—N2—N1	112.93 (16)	C5—C4—C3	120.93 (18)
C1—N2—H2	127.2 (16)	C5—C4—H4A	119.5
N1—N2—H2	119.0 (16)	C3—C4—H4A	119.5
C1—N3—C2	108.48 (15)	C4—C5—C6	120.17 (18)
C1—N3—N4	124.53 (15)	C4—C5—H5	119.9
C2—N3—N4	126.90 (16)	C6—C5—H5	119.9
N3—N4—H4	105.8 (19)	O1—C6—C5	118.31 (17)
N3—N4—H40	109.5 (19)	O1—C6—C7	121.86 (17)
H4—N4—H40	104 (3)	C5—C6—C7	119.83 (17)
O2—C1—N2	128.18 (18)	C8—C7—C6	119.58 (18)
O2—C1—N3	127.92 (18)	C8—C7—H7	120.2
N2—C1—N3	103.90 (16)	C6—C7—H7	120.2
N1—C2—N3	109.81 (15)	C7—C8—C3	121.49 (18)
N1—C2—C3	121.80 (16)	C7—C8—H8	119.3
N3—C2—C3	128.39 (16)	C3—C8—H8	119.3
C4—C3—C8	117.99 (17)		
			
C2—N1—N2—C1	0.4 (2)	N3—C2—C3—C4	−0.2 (3)
N1—N2—C1—O2	179.29 (19)	N1—C2—C3—C8	1.5 (3)
N1—N2—C1—N3	−0.6 (2)	N3—C2—C3—C8	−179.08 (18)
C2—N3—C1—O2	−179.4 (2)	C8—C3—C4—C5	−0.3 (3)
N4—N3—C1—O2	−2.7 (3)	C2—C3—C4—C5	−179.17 (18)
C2—N3—C1—N2	0.5 (2)	C3—C4—C5—C6	0.9 (3)
N4—N3—C1—N2	177.19 (19)	C4—C5—C6—O1	178.66 (18)
N2—N1—C2—N3	−0.1 (2)	C4—C5—C6—C7	−1.4 (3)
N2—N1—C2—C3	179.42 (17)	O1—C6—C7—C8	−178.87 (18)
C1—N3—C2—N1	−0.2 (2)	C5—C6—C7—C8	1.2 (3)
N4—N3—C2—N1	−176.86 (19)	C6—C7—C8—C3	−0.5 (3)
C1—N3—C2—C3	−179.71 (18)	C4—C3—C8—C7	0.1 (3)
N4—N3—C2—C3	3.7 (3)	C2—C3—C8—C7	179.05 (18)
N1—C2—C3—C4	−179.6 (2)		

### Hydrogen-bond geometry (Å, °)

**Table:**

*D*—H···*A*	*D*—H	H···*A*	*D*···*A*	*D*—H···*A*
O1—H1···O2^i^	0.86 (1)	1.78 (1)	2.633 (2)	175 (3)
N2—H2···O1^ii^	0.86 (1)	1.93 (1)	2.789 (2)	173 (2)
N4—H4···O2^iii^	0.87 (1)	2.24 (1)	3.077 (3)	163 (2)

Symmetry codes: (i) *x*, *y*−1, *z*−1; (ii) *x*+1, *y*+1, *z*+1; (iii) −*x*+1, −*y*+2, −*z*+2.
